# Introgression of the *crtRB1* gene into quality protein maize inbred lines using molecular markers

**DOI:** 10.1007/s11032-015-0349-7

**Published:** 2015-07-16

**Authors:** Li Liu, Daniel Jeffers, Yudong Zhang, Meiling Ding, Wei Chen, Manjit S. Kang, Xingming Fan

**Affiliations:** Institute of Food Crops, Yunnan Academy of Agricultural Sciences (YAAS), Kunming, 650205 Yunnan Province China; Yunnan Tian Rui Seed Company, LTD., Kunming, 650205 Yunnan Province China; CIMMYT Yunnan, Institute of Food Crops, Yunnan Academy of Agricultural Sciences (YAAS), Kunming, 650205 Yunnan Province China; Department of Plant Pathology, Kansas State University, Manhattan, KS 66506-5502 USA

**Keywords:** Provitamin A, *crtRB1* gene, Functional markers, Foreground selection, Background selection, Maize (*Zea mays* L.)

## Abstract

Quality protein maize (QPM; *Zea mays* L.) has effectively enhanced levels of the amino acids, lysine, and tryptophan, over normal maize and provided balanced dietary protein for the health and development of monogastric animals and humans. However, as in normal maize, QPM varieties are low in provitamin A (ProVA), a precursor of vitamin A, which can lead to vitamin A deficiency in humans when maize is a significant part of their diet. In this study, maize inbred Hp321-1 carrying the favorable alleles *crtRB1*-*5′TE*-*2* and *crtRB1*-*3′TE*-*1* that can enhance levels of ProVA, was used as donor for improving ProVA in QPM inbred lines CML161 and CML171. Functional markers for identifying the favorable alleles *crtRB1*-*5′TE*-*2* and *crtRB1*-*3′TE*-*1* were used in foreground selection, and simple sequence repeat markers were used in background selection for the BC_1_F_1_, BC_2_F_1_, and BC_2_F_2_ generations. The background recovery rates were 77.4 and 84.5 % for CML161 and CML171 populations, respectively, in the BC_1_F_1_ generation, and 89.9 and 92.1 % in the BC_2_F_2_ generation. With foreground and background selection, the mean ProVA concentration has been improved from 1.60 µg g^−1^ in the parent of CML161 to 5.25 µg g^−1^ in its BC_2_F_3_ offspring, from 1.80 µg g^−1^ in the parent of CML171 to 8.14 µg g^−1^ in its BC_2_F_3_ offspring while maintaining similar QPM characteristics of the recurrent parents. The success from this study offers maize breeders a procedure for increasing ProVA in QPM lines, which will greatly mitigate vitamin A deficiency and protein-energy malnutrition in developing countries.

**Electronic supplementary material:**

The online version of this article (doi:10.1007/s11032-015-0349-7) contains supplementary material, which is available to authorized users.

## Introduction

Maize is the staple food and main source of energy for millions of poor people living in developing countries, including China (especially Southwestern China), India, and many countries in Africa and Latin America (IITA 2009; Nuss and Tanumihardjo 2010; Li et al. 2012). As most of the maize varieties do not contain enough provitamin A (ProVA), the precursor that leads to the formation of vitamin A, people heavily relying on maize diet may have health problems, such as malnutrition, specifically vitamin A deficiency (VAD) (IITA 2009). The VAD may retard growth, increase risk of diseases (e.g., macular degeneration), and cause reproductive disorders in humans (West 1991; Aguayo and Baker 2005; West and Darnton-Hill 2008). Fortunately, maize germplasm has tremendous genetic diversity for ProVA content. There are germplasm available with high levels of ProVA carotenoids, including α-, β-carotene and γ-cryptoxanthin (Pixley et al. 2013). Thus, breeding maize for increased levels of ProVA would be an economical and efficient way to address VAD, especially in the developing world (Yan et al. 2010; Zhang et al. 2012).

ProVA usually accounts for only 10–20 % of the total carotenoids in the maize kernel, and most yellow maize hybrids have <2 μg g^−1^ of ProVA (Ortiz-Monasterio et al. 2007; Pixley et al. 2013). A preliminary target level of 15 μg g^−1^ for ProVA in maize has been set for the HarvestPlus project. This target level of ProVA has been based on three assumptions: (1) gross daily intake of 400 g for adults and 200 g for children; (2) bioavailability ratio of 12:1 μg (retinol activity equivalent); and (3) 50 % retention after processing (Bouis et al. 2011). Following many years of efforts toward increasing ProVA concentrations in maize hybrids, some elite inbreds and maize breeding stocks with substantially high ProVA levels have been developed by plant breeders via the biofortification method, with some levels reaching 15–20 µg g^−1^ ProVA (Babu et al. 2013).

ProVA carotenoids in maize kernels may lead to different colors in the endosperm, varying from light yellow to dark orange (Weber 1987). However, there is a low correlation between visual grain color and total carotenoids, β-carotene, and β-cryptoxanthin in diverse inbreds, and screening for high ProVA concentration based on kernel color is not considered reliable (Harjes et al. 2008; Mishra and Singh 2010). Visible light range (400–1100 nm)-based methods, such as near-infrared reflectance spectroscopy (NIRS), have successfully been applied to detect total carotenoid and major carotenoids, such as lutein and zeaxanthin, but not ProVA carotenoid concentrations (Berardo et al. 2004). Another method used for measuring ProVA is high-performance liquid chromatography (HPLC). Though it has been used widely for measuring ProVA concentrations, it is expensive, time-consuming, and has low throughput, limiting its use for routine screening in conventional maize breeding programs (Pfeiffer and McClafferty 2007).

The ProVA carotenoid biosynthesis pathway is well studied in plants, and the key genes controlling critical steps in the pathway have been successfully identified (Hirschberg 2001; DellaPenna and Pogson 2006; Giuliano et al. 2008; Cazzonelli and Pogson 2010; Farré et al. 2010). In maize, five genes play important roles in the final accumulation of ProVA in the grain. One gene encoding phytoene synthase (*PSY1*) with two favorable alleles is associated with variation in total carotenoids (Fu et al. 2013b). Another gene encoding lycopene epsilon cyclase (*lcyE*) with four favorable alleles is associated with variation in the ratio of various carotenoids in the α- to β-branches of the carotenoid biosynthesis pathway (Harjes et al. 2008). A third gene, *crtRB3*, encodes α-carotene hydroxylase enzyme and a fourth gene, *ZEP1*, controls zeaxanthin epoxidase; both of these genes have been known to play a role in the carotenoid metabolic pathway (Vallabhaneni and Wurtzel 2009; Zhou et al. 2012). A fifth gene encodes β-carotene hydroxylase enzyme (*crtRB1*) with three favorable alleles and it has a significant impact on variation for β-carotene concentration in the endosperm (Fu et al. 2013a). Yan et al. (2010) found that ProVA concentrations of haplotypes with *CrtRB1*-*5′TE* and *CrtRB1*-*3′TE* favorable alleles were 5.2-fold higher than those of all other haplotypes. Babu et al. (2013) further reported that *CrtRB1* clearly had a much larger effect on ProVA concentration than *LcyE*. However, limited information is available about applying the marker-assisted backcross (MAB) method to select favorable alleles of the *CrtRB1* gene for increasing ProVA concentrations in maize grain.

Marker-assisted selection (MAS) is regarded as a key method for increasing ProVA concentrations in maize (Prasanna et al. 2010). Two genes, *CrtRB1* on chromosome 10 (Yan et al. 2010) and *LcyE* on chromosome 8 (Harjes et al. 2008), have the most significant effect on ProVA concentrations in maize grain (Babu et al. 2013). The effectiveness of molecular marker polymorphisms in linking *LcyE* and *CrtRB1* to ProVA concentrations has been verified using 26 tropical maize populations, and the functional gene markers for high ProVA concentration have been used in MAS (Azmach et al. 2013). As Benchimol et al. (2005) pointed out that through a backcross breeding program, source genes related to high ProVA concentration can be integrated into genotypes with elite agronomic traits of the recurrent parents. However, one of the major limitations is the long period of time required for the backcross procedure. Therefore, molecular markers are important tools for accelerating the recovery of recurrent parent genome as well as assisting in the selection of plants that carry a desired marker linked to high ProVA concentration (Bouchez et al. 2002; Oliveira et al. 2008). Marker-assisted backcrossing is highly suited to monitoring the degree of similarity of the lines to the recurrent parent.

The QPM maize inbred lines CML161 and CML171 have been derived from the CIMMYT G25QPM germplasm and have been widely used in genetic studies and in several breeding programs (Fan et al. 2001; Amiruzzaman et al. 2011; Setter et al. 2011; Fan et al. 2014). The maize commercial hybrids HQ-2000 (Vietnam), INIA (Peru), and Yunyou 78 (China) have been developed utilizing CML161 (Prasanna et al. 2001; Fan et al. 2006); other commercial hybrids including Yunrui 8, Yunyou 89, Qiandan 11, and Quian 2609 (China) utilize CML171 (Cordova 2001; Zhu et al. 2003; Fan et al. 2014). These released hybrids have played a very important role in alleviating the malnutrition of children and have been used as an alternative feed ration for swine and poultry, where conventional sources of lysine are from soybean meal or synthetic lysine (López-Pereira 1992). The integration of the favorable alleles *crtRB1*-*5′TE*-*2* and *crtRB1*-*3′TE*-*1* for the *crtRB1* gene for high ProVA concentration into the widely utilized QPM maize lines CML161 and CML171 would provide a valuable nutritional enhancement to the grain for use in the resource-poor regions of the world. The objectives of this study were: (1) to increase ProVA concentrations of QPM maize lines CML161 and CML171 via MAB using a maize line as donor that carries two favorable alleles of *CrtRB1*, i.e., *crtRB1*-*5′TE*-*2* and *crtRB1*-*3′TE*-1, for high ProVA concentrations and (2) to evaluate the effectiveness of the MAB procedure for transferring the target genes into local elite maize lines by examining the recovery rates of the recurrent parents.

## Materials and methods

### Plant materials

The temperate maize inbred line Hp321-1 (provided by Prof. Jianbing Yan, Huazhong Agricultural University) carrying the favorable alleles *crtRB1*-*5′TE*-*2* and *crtRB1*-*3′TE*-*1* of the *crtRB1* gene (ProVA concentration = 9.74 µg g^−1^), was used as the donor parent (male). Two tropical QPM maize inbred lines, CML161 and CML171, were used as the recurrent female parents. At the *lcyE* locus, there is no polymorphism among Hp321-1, CML161, and CML171; thus gene pyramiding was not involved.

### Foreground selection

Foreground selection (FS) refers to selection of favorable alleles (*crtRB1*-*5′TE*-*2* and *crtRB1*-*3′TE*-*1*) for higher ProVA concentration. Polymerase chain reaction (PCR) amplification of the functional marker for *crtRB1*-*5′TE*-*2* was done using the forward primer 5′-TTAGAGCCTCGACCCTCTGTG-3′ and the reverse primer 5′-AATCCCTTTCCATGTTACGC-3′. For *crtRB1*-*3′TE*-*1*, the forward primer was 5′-ACACCACATGGACAAGTTCG-3′ and the reverse primers were 5′-ACACTCTGGCCCATGAACAC-3′ and 5′-ACAGCAATACAGGGGACCAG-3′ (Yan et al. 2010).

### Background selection

Background selection (BS) refers to selection of the genetic background of the individuals selected via FS. Two DNA bulks were constructed by mixing equal amounts of DNA from five plants of the donor parent (Hp321-1) and five plants of each recurrent parent CML161 and CML171 for screening parental polymorphic SSR markers. In total, 760 SSRs were retrieved from the Maize Genetics and Genomics Database (http://www.maizegdb.org/) and synthesized by Sangon Biotech (Shanghai, China). A total of 98 polymorphic SSR markers between Hp321-1 and CML161, and 89 polymorphic SSR markers between Hp321-1 and CML171, evenly distributed on 10 maize chromosomes, were used for genotyping the FS-selected individuals of the BC_1_F_1_, BC_2_F_1_, and BC_2_F_2_ populations developed with CML161 and CML171.

### Backcross breeding program

A MAB breeding program was used to integrate the favorable alleles *crtRB1*-*5′TE*-*2* and *crtRB1*-*3′TE*-*1* from the temperate inbred line Hp321-1 into the tropical QPM maize inbred lines CML161 and CML171, for increasing ProVA concentration. The population development involved two parallel crossing schemes. The QPM inbred line was crossed to Hp321-1 to produce F_1_, and then backcrossed to CML161 two times to produce BC_1_F_1_ and BC_2_F_1_. Self-pollination was made to produce BC_2_F_2_ and BC_2_F_3_ populations. The F_1_, BC_1_F_1_, BC_2_F_1_, BC_2_F_2_, and BC_2_F_3_ populations of CML 171 were produced using the same crossing schemes. In the summer 2010, seeds of CML161, CML171, and Hp321-1 were sown and crosses were made at the YAAS Baiyi Maize Research Station (BMRS, 25°23′N, 102°9′E, 1970 MASL), Kunming, Yunnan, China, to generate F_1_ populations of CML161 and CML171. In the winter 2010, the F_1_ was used to develop ‘backcross progenies’ (BC_1_F_1_ populations) by backcrossing the F_1_ with their recurrent parents CML161 and CML171 at the YAAS Gasa Maize Research Station (GMRS, 21°95′N, 100°8′E, 588 MASL), Jinghong, Yunnan, China. In the summer 2011, 597 BC_1_F_1_ plants of the CML161 population and 462 BC_1_F_1_ plants of the CML171 population were selected via FS and then screened with BS for recovering individuals with ≥80.0 % genetic similarity to the recurrent parent to develop ‘backcross progenies’ BC_2_F_1_ populations by backcrossing the BC_1_F_1_ with their recurrent parent CML161 and CML171 at the BMRS. In the winter 2011, 779 BC_2_F_1_ plants of the CML161 population and 1055 BC_2_F_1_ plants of the CML171 population selected via FS were further used to develop ‘selfed progeny’ BC_2_F_2_ at the GMRS. In the summer 2012, 1428 BC_2_F_2_ plants of the CML161 population and 1554 BC_2_F_2_ plants of the CML171 population were planted at the BMRS, and 2452 BC_2_F_2_ plants of the CML161 population and 2775 BC_2_F_2_ plants of the CML171 population were planted at the Yanshan Maize Research Station (YMRS, 23°60′N, 104°4′E, 1570 MASL), Yanshan, Yunnan, China. Each BC_2_F_2_ plant of CML161 population and CML171 population at both locations was selected via FS and then screened for a recovery rate of ≥90.0 % of the recurrent parent via BS, to generate BC_2_F_3_ for phenotyping for ProVA concentration, lysine and tryptophan contents. The two parallel crossing schemes for CML161 and Hp321-1, and CML171 and Hp321-1 are shown in Supplementary Fig. 1.

### Genotyping

A modified CTAB method (Dellaporta et al. 1983) was used for DNA extraction. The reaction for genotyping with the two functional markers in FS consisted of a total volume of 15 μl containing 30 ng genomic DNA, 0.2 μM of each primer, 1.5 μl of 10× *Taq* DNA polymerase buffer (20 mM MgCl_2_), 0.1 mM of each dNTPs (TransGen Biotech, China), and l U of *Taq* DNA polymerase (TransGen Biotech, China). For parental polymorphic SSR markers in BS, a modified PCR reaction according to McCouch et al. (2002) was used. The reaction consisted of a total volume of 10 μl containing 20 ng genomic DNA, 0.3 μM of each primer, 1.0 μl of 10× *Taq* DNA polymerase buffer (20 mM MgCl_2_), 0.1 mM of each dNTPs (TransGen Biotech, China), and l U of *Taq* DNA polymerase (TransGen Biotech, China).

A modified PCR program (touchdown PCR) was used in profiling functional markers for the favorable alleles (*crtRB1*-*5′TE*-*2* and *crtRB1*-*3′TE*-*1*) in plants to avoid the amplification of nonspecific, spurious PCR products. The reaction profile with Mastercycler^®^ gradient (Eppendorf, Germany) was performed using the following protocol: First initial hold was at 95 °C for 5 min. The second hold starting with denaturation step at 95 °C for 1 min, annealing at 64 °C for 1 min (10 cycles for *crtRB1*-*5′TE*-*2*, and 19 cycles for *crtRB1*-*3′TE*-*1*, reducing 0.5 °C per cycle), and the extension step at 72 °C for 1 min. The third hold starting at a denaturation temperature of 95 °C for 1 min, annealing at 55 °C for 1 min, and extension at 72 °C for 1 min with 27 cycles for *crtRB1*-*5′TE*-*2* and 19 cycles for *crtRB1*-*3′TE*-*1*. The final extension step was done at 72 °C for 10 min. The amplified fragments were resolved in a 2 % agarose gel for analyzing the amplicons.

In profiling of polymorphic SSR markers in BS, the reaction profile was 5 min at 94 °C with 34 cycles of 45 s at 95 °C, and 45 s at 57 °C annealing, and 1 min at 72 °C and 10 min at 72 °C for final extension. The PCR products were separated via electrophoresis on 6 % polyacrylamide gel.

### ProVA concentration estimation in parents and the bulked BC_2_F_3_ populations

ProVA concentration was estimated according to the modified method of Kimura and Rodrigeuz (2002). A 20-kernel sample was collected from the middle of the ear on each individual plant sampled from the BC_2_F_3_ population with the favorable alleles (*crtRB1*-*5′TE*-*2* and *crtRB1*-*3′TE*-*1*) and from the parental inbreds CML161 and CML171 per se. A total of 127 and 145 ears were sampled from the CML161 and CML171 BC_2_F_3_ populations at the BMRS, and 158 and 133 ears were sampled from the respective BC_2_F_3_ populations at the YMRS. Five ears were sampled from CML161 and CML171. All samples were mixed and ground with a Cyclotec™ 1093 (FOSS TECATOR) sample mill. A 0.5-g sample of ground grain was placed in the extraction tube, 6 ml of ethanol plus 0.1 % butylated hydroxyl toluene was added, mixed and incubated at 85 °C for 5 min, followed by the addition of 500 μl of 80 % potassium hydroxide (w/v) and incubated at 85 °C for 10 min. After saponification, 3 ml of cold dH_2_O was added, followed by incubation on ice. A 200 μl internal standard of β-Apo-8′-carotenal and 4 ml hexane were added. The sample was vortexed and centrifuged at 1200×*g,* and the top hexane layer was transferred to a new tube. The remaining aqueous layer was extracted twice with 3 ml hexane. The combined hexane layers were freeze-dried and stored at −85 °C. Prior to HPLC injection, the carotenoids were reconstituted in 500 μl of acetonitrile:methanol:methylene chloride (45:20:35). All extraction procedures were repeated three times from the ground samples, and each extract was used in separated HPLC analysis.

Carotenoids were separated by HPLC (Agilent-1200) at DL (Shanghai) Company Limited using an YMC CT99S05-2546WT C30 Carotenoid Column. One hundred microliters of the sample was loaded into glass injection vials, and 50 μl was injected at a flow rate of 1.8 ml min^−1^. The mobile phase consisted of acetonitrile:methanol:methylene chloride at 75:20:4, containing 0.05 % triethylamine (TEA) and 0.1 % BHT. A multi-wavelength detector was set at 450 nm and used to detect the absorbance of carotenoids. ProVA concentration (μg g^−1^ of dry matter) was calculated as the sum of β-carotene and half of each of β-cryptoxanthin and α-carotene concentrations.

### Lysine and tryptophan analysis in parents and the bulked BC_2_F_3_ populations

Lysine and tryptophan concentrations were estimated according to the method of Galicia et al. (2008) for a 20-kernel sample from each individual BC_2_F_3_ with the favorable alleles (*crtRB1*-*5′TE*-*2* and *crtRB1*-*3′TE*-*1*), and the parents. After grinding and defatting treatment, 100 mg of the powder for the lysine test was placed in 15 ml extraction tubes, and 3 ml papain solution was added, then mixed and incubated at 64 °C for 16 h. The samples were centrifuged at 2500 rpm for 5 min, and 1 ml of the supernatant was transferred to a new tube and 0.1 ml of 2-chloro-3,5-dinitropyridine reagent (Sigma-Aldrich, America) was added. The mixture was incubated for 2 h at room temperature and then 5 ml of 1.2 N HCl and 5 ml of ethyl acetate were added. The upper phase was removed, and the absorbance was measured by a spectrophotometer (HITACHI U3900/3900H, Japan) at 390 nm, and the lysine concentration was calculated using the following formula:

$$\% \,{\text{Lysine}}\,{\text{concentration}}\, (\upmu{\text{g/}}\upmu{\text{g)}} = \frac{A}{\text{slope}} \times \frac{{{\text{hydrolysis}}\,{\text{volume}}}}{{{\text{sample}}\,{\text{weight}}}} \times 100\,\%$$

where *A* = absorbance at 390 nm.

After the grinding and defatting treatment, 80 mg of the powder for the tryptophan test was placed in a 15-ml extraction tube, and 5 ml of papain solution was added, then mixed, and incubated at 64 °C for 16 h. The samples were centrifuged at 3600×*g* for 5 min; 1 ml of the supernatant was transferred to a glass tube, and 3 ml of the colorimetric reagent (Sigma-Aldrich, America) was added. The sample was mixed and incubated at 64 °C for 30 min, and the absorbance was measured by a spectrophotometer (HITACHI U3900/3900H, Japan) at 560 nm, and the tryptophan concentration was calculated using the following formula:

$$\% \,{\text{Tryptophan}}\,{\text{concentration}}\, (\upmu{\text{g/}}\upmu{\text{g)}} = \frac{A}{\text{slope}} \times \frac{{{\text{hydrolysis}}\,{\text{volume}}}}{{{\text{sample}}\,{\text{weight}}}} \times 100\,\%$$

where *A* = absorbance at 560 nm.

### Statistical analysis

The gel bands were scored as A for the P_1_ functional allele (named as genome donor, GD), B for the P_2_ functional allele (named as genome recurrent, GR), H for the F_1_ pattern of P_1_ and P_2_ functional alleles, and U for an unidentified band. The percentage of the P_1_ recurrent parent genome present in each genotyped plant was estimated using the expression GR% = [*B* + (0.5*H*)/(*B* + *H* + *A*)] × 100 %, corresponding to the number of *B* functional alleles present in the genotype of each genotyped plant (Benchimol et al. 2005). The introgressed locus was taken from the computation of the total recovery of recurrent parent genotypes. Analysis of variance and calculation of standard deviation were performed using SAS 9.1 software (SAS Institute Inc 2004) for ProVA, lysine, and tryptophan concentration in the BC_2_F_3_ individuals, and the recovery rate of the recurrent parent following BS.

## Results

### Polymorphism of functional markers and SSR markers between parents

The two functional markers of *crtRB1* gene were polymorphic between the donor parent (Hp321-1) and the recurrent parents CML161 and CML171 (Supplementary Fig. 2). The donor parent Hp321-1 had the favorable allele *crtRB1*-*5′TE*-*2* with a 600-bp band, while unfavorable allele *crtRB1*-*5′TE*-*1* had an 800-bp band corresponding to the recurrent parents CML161 and CML171. Furthermore, the donor parent Hp321-1 carried the favorable allele *crtRB1*-*3′TE*-*2* with a 543-bp band, while the recurrent parents exhibited the unfavorable allele *crtRB1*-*3′TE*-*2* with a 296 + 875-bp band in CML161, and *crtRB1*-*3′TE*-*3* with a 296 + 1221-bp band in CML171. The polymorphic markers were codominant, which rendered them usable for the MAB of the corresponding target genes. Therefore, the functional markers of favorable alleles (*crtRB1*-*5′TE*-*2* and *crtRB1*-*3′TE*-*1*) were used for FS selection.

In total, 760 SSR markers distributed across the 10 chromosomes of the maize genome were selected for the screening of polymorphisms between the three parent lines. Of these markers, 98 polymorphic SSR markers (12.9 %) between Hp321-1 and CML161, and 89 polymorphic SSR markers (11.7 %) between Hp321-1 and CML171 were identified for BS selection.

### Success in transferring the favorable alleles from the donor to recurrent parents

The two recipient parents, CML161 and CML171, were used as recurrent parents to cross to the donor parent, Hp321-1, which had two favorable alleles (*crtRB1*-*5′TE*-*2* and *crtRB1*-*3′TE*-*1*) for high ProVA concentrations to produce the F_1_, BC_1_F_1_, BC_2_F_1_, and BC_2_F_2_ generations. In the segregating generations, i.e., BC_1_F_1_, BC_2_F_1_, and BC_2_F_2_, both favorable alleles (*crtRB1*-*5′TE*-*2* and *crtRB1*-*3′TE*-*1*) were detected (Table 1). The results showed that *crtRB1*-*5′TE*-*2* and *crtRB1*-*3′TE*-*1* alleles had linkage in F_1_ and BC_1_F_1_ generations, and no linkage in BC_2_F_2_ generation.

**Table 1:** Foreground selection for BC_1_F_1_, BC_2_F_1_ and BC_2_F_2_ generations in the CML161 and CML171 populations using functional markers

Inbred	Generation	Location	Replicate	Total plants	Plant with homozygous site	Plant with heterozygous site	Plant without target site	*P* value (*χ* ^2^ test)
CML161	BC_1_F_1_	–	–	597	290	–	307	0.4866
	BC_2_F_1_	–	–	779	410	–	369	0.1418
	BC_2_F_2_	BMRS	1	737	187	374	176	0.4161
			2	691	153	364	174	
		YMRS	1	1268	322	588	358	0.0001
			2	1184	307	541	336	
CML171	BC_1_F_1_	–	–	462	218	–	244	0.2264
	BC_2_F_1_	–	–	1055	497	–	558	0.0604
	BC_2_F_2_	BMRS	1	702	162	391	149	0.0002
			2	852	201	466	185	
		YMRS	1	1401	358	655	388	0.0226
			2	1374	373	660	341	

In the BC_1_F_1_ generation, 597 plants of the CML161 population and 462 plants of the CML171 population were selected and FS was performed for the target alleles (Supplementary Fig. 3). A total of 290 individuals carried the favorable alleles *crtRB1*-*5′TE*-*2* and *crtRB1*-*3′TE*-*1* in the CML161 population, and 218 individuals in the CML171 population. In the BC_2_F_1_ generation, 779 plants of the CML161 and 1055 plants of the CML171 were chosen, and FS was applied for the two target favorable alleles (Supplementary Fig. 4). A total of 410 individuals had the favorable alleles *crtRB1*-*5′TE*-*2* and *crtRB1*-*3′TE*-*1* in the CML161 population, and 497 individuals in the CML171 population. Chi-square tests showed that segregation ratios for the favorable allele combination of *crtRB1*-*5′TE*-*2* and *crtRB1*-*3′TE*-*1* in four populations of BC_1_F_1_ and BC_2_F_1_ derived from CML161 and CML171 were a good fit to the Mendelian 1:1 ratio expected for one gene-pair inheritance (*P* > 0.05).

In the BC_2_F_2_ generation, 1428 plants of the CML161 population and 1554 plants of the CML171 population were selected at the BMRS and FS was performed for the target favorable alleles (Supplementary Fig. 5). In total, 738 individuals carried the favorable allelic combination of *crtRB1*-*5′TE*-*2* and *crtRB1*-*3′TE*-*1* in the CML161 population, and 857 individuals in the CML171 population. At the YMRS, 2452 plants of the CML161 population and 2775 plants of the CML171 population were selected and FS was performed for the favorable alleles. In total, 1129 individuals had the favorable allelic combination of *crtRB1*-*5′TE*-*2* and *crtRB1*-*3′TE*-*1* in the CML161 population and 1315 individuals in the CML171 population. Chi-square test showed that segregation ratio for the favorable alleles *crtRB1*-*5′TE*-*2* and *crtRB1*-*3′TE*-*1* in the BC_2_F_2_ population of CML161 was a good fit to the Mendelian 1:2:1 ratio expected for one gene-pair inheritance (*P* > 0.05), while CML171 population at the BMRS did not fit this ratio (*P* < 0.05). At the YMRS, the segregation ratios for the favorable alleles *crtRB1*-*5′TE*-*2* and *crtRB1*-*3′TE*-*1* for both populations (CML161 and CML171) did not fit the Mendelian 1:2:1 ratio (*P* < 0.05).

The results demonstrated that favorable alleles *crtRB1*-*5′TE*-*2* and *crtRB1*-*3′TE*-*1* for high ProVA concentration were successfully transferred into the two QPM maize lines. The two favorable alleles, *crtRB1*-*5′TE*-*2* and *crtRB1*-*3′TE*-*1*, displayed a 1:1 Mendelian ratio in F_1_ and BC_1_F_1_ generation and segregated in an approximately 1:2:1 ratio in BC_2_F_2_ generation.

### Improvement in the level of ProVA in kernel of QPM maize lines

**Fig. 1:**
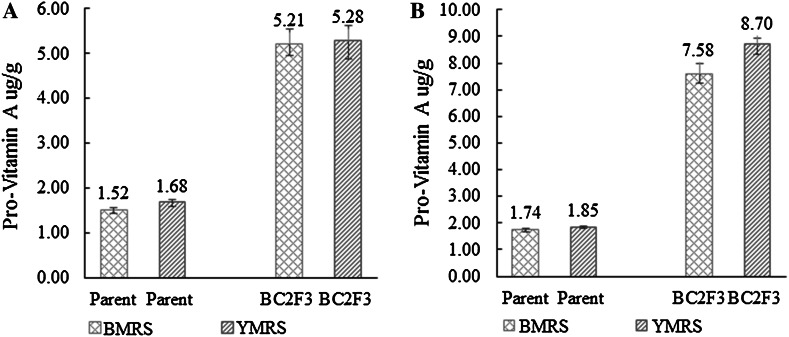
Increase in ProVA concentrations in the QPM maize through molecular marker-assisted foreground and background selections. **a** The increase in ProVA concentration in the CML161 population. **b** The increase in ProVA concentration in the CML171 population

In total, the seeds from 127 and 145 plants of BC_2_F_3_ generation of CML161 and CML171 populations with the favorable allele combination of *crtRB1*-*5′TE*-*2* and *crtRB1*-*3′TE*-*1* at the BMRS, and 158 and 133 plants of CML161 and CML171 populations with the favorable allele combination of *crtRB1*-*5′TE*-*2* and *crtRB1*-*3′TE*-*1* at the YMRS were sampled. The ProVA concentrations of the bulked BC_2_F_3_ populations were tested using HPLC, and the results are presented in Fig. 1. The mean ProVA concentrations of the BC_2_F_3_ generation of CML161 population at the BMRS and YMRS stations were 5.21 and 5.28 µg g^−1^, with a mean of 5.25 µg g^−1^, which was significantly higher than that of recurrent parent CML161, with ProVA concentrations of 1.52 and 1.68 µg g^−1^, with a mean of 1.60 µg g^−1^, respectively (*P* < 0.01). Similar results were obtained for the second parallel population of CML171. The mean ProVA concentrations of the BC_2_F_3_ generation of CML171 population at the BMRS and YMRS stations were, respectively, 7.58 and 8.70 µg g^−1^, with a mean of 8.14 µg g^−1^, which was significantly higher than that of recurrent parent CML171, with the ProVA concentrations of 1.74 and 1.85 µg g^−1^, with a mean of 1.80 µg g^−1^, respectively (*P* < 0.01). The results showed that QPM inbred lines CML161 and CML171 introgressed with the favorable alleles *crtRB1*-*5′TE*-*2* and *crtRB1*-*3′TE*-*1* had 3.28-fold and 4.52-fold increase in the mean ProVA concentration, which indicated that the MAB procedure was effective in transferring the favorable *crtRB1* gene and increasing the ProVA level in maize. Generally, the level of ProVA at the YMRS tended to be higher than that at the BMRS; however, the difference was not significant. The results indicated that ProVA concentrations of maize might be somewhat affected by genotype x environment interaction, but the ProVA was mainly controlled by genetic factors.

### Effectiveness of the marker-assisted backcross procedure

**Fig. 2:**
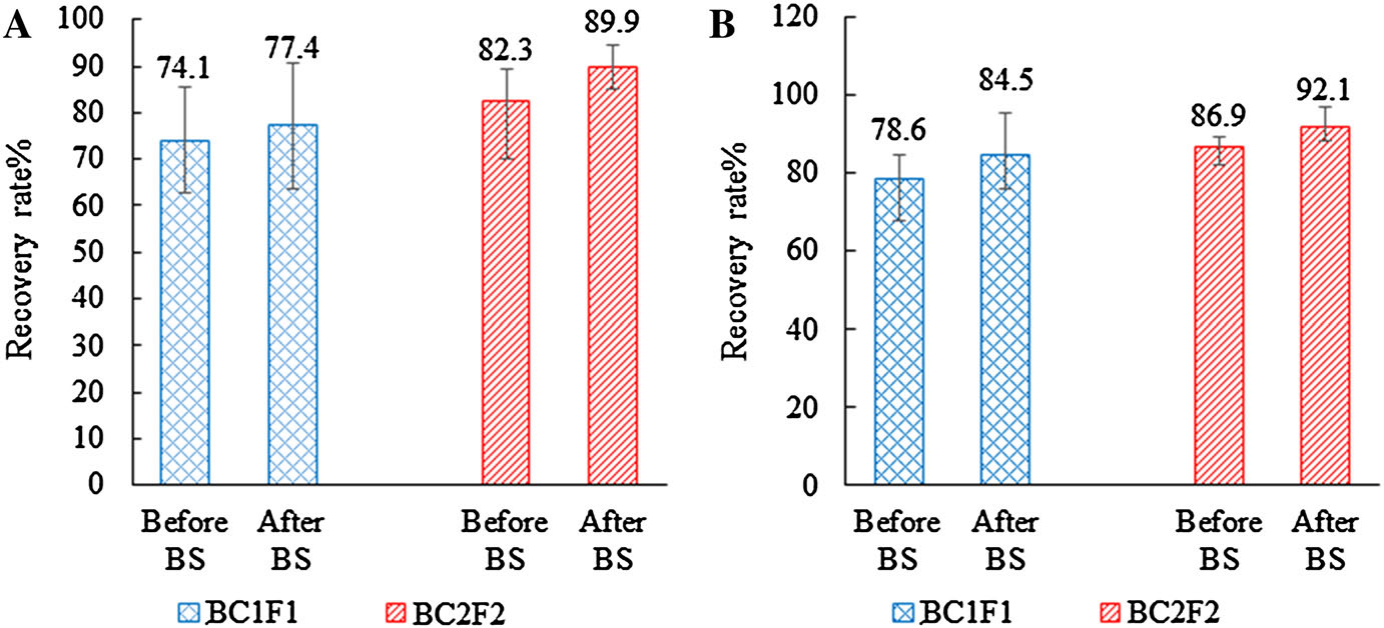
Recovery rate before and after background selections (BS) in BC_1_F_1_ and BC_2_F_2_ generations. **a** The recovery rate in the CML161 populations. **b** The recovery rate in the CML171 populations

Among 760 SSR primers surveyed, 98 polymorphic markers between CML161 and HP321-1, and 89 polymorphic markers between CML171 and HP321-1 were used for selection in each segregating generation, i.e., BC_1_F_1_ and BC_2_F_2_ (Table 2) and the results presented as genetic background recovery rate in Fig. 2. In the BC_1_F_1_ generation, 276 plants from the CML161 population were tested and the results showed that the genetic background recovery rate of selected individuals varied from 63.8 to 90.8 %, with a mean of 77.4 %. This was 3.3 % higher than the mean recovery rate of 74.1 %, varied from 62.7 to 85.5 %, before BS; 210 plants from the CML171 population were analyzed and the results indicated that the genetic background recovery rate of selected individuals ranged from 75.8 to 95.5 %, with a mean of 84.5 %. This was 5.9 % higher than the mean recovery rate of 78.6 %, varied from 67.9 to 84.6 %, before BS. The plants in both populations with a recovery rate of ≥80.0 % were selected for backcrossing.

**Table 2:** Background selection for BC_1_F_1_ and BC_2_F_2_ generations of CML161 and CML171 populations using SSR markers

Generation	Population	Site of *crtRB1*	Number of plants	Number of polymorphic markers	Range of recovery rate (%)	Mean recovery rate (%)
BC_1_F_1_	CML161	*crtRB1*-*5′TE*-*2* and *crtRB1*-*3′TE*-*1*	276	98	63.8–90.8	77.4
	CML171	*crtRB1*-*5′TE*-*2* and *crtRB1*-*3′TE*-*1*	210	89	75.8–95.5	84.5
BC_2_F_2_	CML161	*crtRB1*-*5′TE*-*2*	187	98	81.9–87.2	84.8
		*crtRB1*-*5′TE*-*2* and *crtRB1*-*3′TE*-*1*	374	98	85.1–94.7	89.9
		*crtRB1*-*3′TE*-*1*	176	98	84.0–96.8	90.4
	CML171	*crtRB1*-*5′TE*-*2*	162	89	80.9–90.4	84.8
		*crtRB1*-*5′TE*-*2* and *crtRB1*-*3′TE*-*1*	391	89	88.3–96.8	92.1
		*crtRB1*-*3′TE*-*1*	149	89	90.4–97.9	93.6

In the BC_2_F_2_ generation, 737 plants from CML161 were tested and the results showed that the genetic background recovery rate of selected individuals with the combination of favorable alleles *crtRB1*-*5′TE*-*2* and *crtRB1*-*3′TE*-*1* varied from 85.1 to 94.7 %, with a mean of 89.9 %. This was 7.6 % higher than the mean recovery rate of 82.3 %, varied from 70.1 to 89.2 %, before BS; 702 plants from CML171 were tested and the results indicated that the genetic background recovery rate of selected individuals with the combination of favorable alleles *crtRB1*-*5′TE*-*2* and *crtRB1*-*3′TE*-*1* ranged from 88.3 to 96.8 %, with a mean of 92.1 %. This was 5.2 % higher than the mean recovery rate of 86.9 %, varied from 82.4 to 89.5 %, before BS. The results from both populations showed that the individuals of two consecutive backcross generations using molecular MAS had attained similar levels of homozygosity for the favorable alleles of the recurrent parent. The plants carrying favorable alleles *crtRB1*-*5′TE*-*2* and *crtRB1*-*3′TE*-*1* in both populations with a recovery rate of ≥90.0 % were selected to generate the BC_2_F_3_ generation.

### Levels of lysine and tryptophan in the backcross-derived integrated lines

**Fig. 3:**
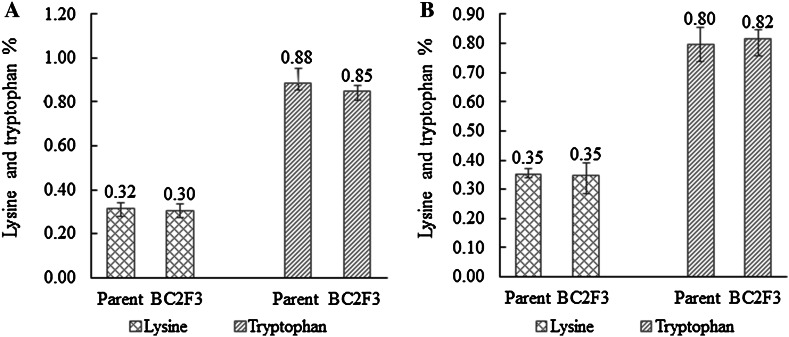
Levels of lysine and tryptophan in backcross-derived lines through molecular marker-assisted foreground and background selections. **a** The levels of lysine and tryptophan in the CML161 population. **b** The levels of lysine and tryptophan in the CML171 population

To determine whether the QPM characteristics of the populations were maintained, seeds from BC_2_F_3_ generation of CML161 and CML171 populations and from the parental inbreds CML161 and CML171 per se were analyzed for lysine and tryptophan concentration at both locations. The results are presented in Fig. 3. Figure 3 shows that the mean lysine and tryptophan contents are 0.32 and 0.88 % for inbred line CML161, respectively, and the mean lysine and tryptophan contents for CML171 were 0.35 and 0.80 %, respectively, while the mean lysine and tryptophan contents for backcross-derived lines of BC_2_F_3_ from CML161 were 0.30 and 0.85 %, respectively, and the mean lysine and tryptophan contents for backcross-derived lines of BC_2_F_3_ from CML171 were 0.35 and 0.82 %, respectively. There were almost no difference in the lysine and tryptophan contents between the BC_2_F_3_ lines and the original CML161 and CML171 inbred lines. The results suggested that MAS backcross procedure had improved ProVA levels without decreasing lysine and tryptophan levels in QPM maize lines.

## Discussion

The ProVA concentrations of both populations derived from inbred lines CML161 and CML171 at the YMRS were higher than those at the BMRS, while the difference between the two stations was not significant. The enhanced protein quality for the derived populations was also maintained in the backcross-derived lines. The QPM lines with improved ProVA concentration are very important breeding materials for developing QPM maize hybrids with high ProVA for people dependent on maize as a major component of their diet. Therefore, the development and production of QPM hybrids with high ProVA concentrations are an efficient way to deliver biofortified maize for improved health and development benefits to the poor people living in developing countries, such as China (especially Southwestern China), India, and several countries in Africa and Latin America.

Previous investigations have revealed that the differences in expression levels of the two loci *LcyE* and *CrtRB1* were mainly expressed in the endosperm and were not different in embryos (Harjes et al. 2008; Yan et al. 2010). Thus, selecting for favorable mutant alleles of *LcyE* and/or *CrtRB1* could cause undesirable effects in the endosperm. In the present study, MAB technology was used to produce two QPM populations with higher levels of ProVA than those of their parents. In the CML161 and CML171 populations, the mean ProVA levels were up to 5.25 and 8.14 µg g^−1^, respectively, which were significantly higher than those of recurrent parents CML161 (1.60 µg g^−1^) and CML171 (1.80 µg g^−1^) (*P* = 0.01). Meanwhile, the lines maintained their protein quality with a lysine content of 0.30 and 0.35 %, and a tryptophan content of 0.85 and 0.82 % in CML161 and CML171 populations with the ProVA being 3.28-fold and 4.52-fold higher than those of the QPM parents CML161 and CML171, respectively. Though the target concentration of >15 µg g^−1^ for ProVA was not achieved, these lines with enhanced levels of ProVA over their original QPM parent lines could be used as parents to develop maize cultivars that meet the dietary needs of both humans and livestock. These improved QPM lines can be used for developing nutritionally enhanced hybrids. This study is the first report for combining functional marker selection for *CrtRB1*-*5′TE* and *CrtRB1*-*3′TE* favorable alleles with the backcross method to obtain improved ProVA lines while maintaining the lysine and tryptophan level of QPM. The authors hope this effective procedure will be applied by more breeders toward mitigating both VAD and protein-energy malnutrition in developing countries.

The MAS for the *CrtRB1* locus appeared to be a reliable strategy for rapidly achieving genetic gains for high ProVA concentration in previous research (Azmach et al. 2013; Babu et al. 2013). However in this study, both microsatellites and MAB selection were used simultaneously. The functional markers of *CrtRB1* locus were used to identify the favorable alleles *crtRB1*-*5′TE*-*2* and *crtRB1*-*3′TE*-*1* for a rapid tripling of ProVA concentration. Meanwhile, when SSR markers were combined for the BS, more than 90 % of the recurrent parent could be recovered via only two backcrosses, while at least three backcrosses would be required in conventional breeding process (Chen et al. 2010). The results indicated that the MAB procedure was an effective strategy for transferring the target genes into local elite maize lines by examining the recovery rates of the recurrent parents. Therefore, this study provided a good example to increase the efficiency in the breeding program for high ProVA level via the BS with SSR markers.

When the functional markers of *crtRB1*-*3′TE* alleles were used to screen the progenies, we found that the upper bands for the *crtRB1*-*3′TE*-*2/3* favorable allele with 845- and 1221-bp were not always present. For example, very few progeny in the BC_1_F_1_ generation had the 845- and 1221-bp bands for the *crtRB1*-*3′TE*-*2*/*3* favorable allele, while the two bands were frequently displayed in the BC_2_F_2_ generation. Compared with a previous report (Yan et al. 2010), the bands of 845- and 1221-bp were much fainter than the bands of 296- and 543-bp. The possible reason might be that the bands of 845- and 1221-bp required more stringent conditions for the PCR reaction. A similar problem was observed in another study (Babu et al. 2013). Therefore, we recommend that the favorable allele of *crtRB1*-*3′TE* gene should be identified using high-fidelity DNA polymerase and the progenies can be tested by universal DNA polymerase.

In the FS for the favorable alleles *crtRB1*-*5′TE*-*2* and *crtRB1*-*3′TE*-*1* in various segregating generations, we found that at the YMRS, the segregation ratio of BC_2_F_2_ generation for CML161 did not fit the Mendelian 1:2:1 ratio expected for one gene inheritance (*P* < 0.05). Similar results were observed in BC_2_F_2_ generation of CML171 at the BMRS and YMRS. A possible reason for this deviation might be that the progenies were artificially selected to have both favorable alleles (*crtRB1*-*5′TE*-*2* and *crtRB1*-*3′TE*-*1*) or it could be because *crtRB1*-*5′TE*-*2* and *crtRB1*-*3′TE*-*1* occurred in a known segregation distortion region (Lu et al. 2002; Babu et al. 2013). We suggest that a larger population size would be required to resolve segregation distortion effects on selection in the breeding program for high ProVA level via functional markers.

## Electronic supplementary material

Supplementary Fig. 1 General scheme for the development of backcross and selfed progenies. The temperate maize inbred line Hp321-1 with favorable alleles *crtRB1*-*5’TE*-*2* and *crtRB1*-*3’TE*-*1* for high ProVA concentrations was used as male and donor parent. The tropical QPM maize inbred lines CML161 and CML171 were used as female and recurrent parents. The numbers 1, 2, 3, 4, and 5 refer two populations developed for CML161 and CML171, respectively. (TIFF 121 kb)

Supplementary Fig. 2 Electrophoresis pattern of functional markers between parents for target alleles. (A) Patterns of *crtRB1*-*5’TE*-*2* allele; (B) Patterns of *crtRB1*-*3’TE*-*1* allele; 1, DNA marker; 2, Hp321-272; 3, Hp321-3; 4, Hp321-1; 5, Sc55; 6, A619; 7, By804; 8, CML161; 9, CML171; 10, P138. Lanes 2–7 are maize inbred lines with higher ProVA concentration and favorable *LcyE* and *crtRB1* allelic combination. Lanes 8–10 are maize inbred lines with lower ProVA concentration and unfavorable *LcyE* and *crtRB1* allelic combination. (TIFF 195 kb)

Supplementary Fig. 3 Detection of the favorable alleles *crtRB1*-*5’TE*-*2* and *crtRB1*-*3’TE*-*1* in BC_1_F_1_ generation. (A) Patterns of *crtRB1*-*5’TE*-*2* allele in the CML161 population, (B) Patterns of *crtRB1*-*3’TE*-*1* allele in the CML161 population, (C) Patterns of *crtRB1*-*5’TE*-*2* allele in the CML171 population, (D) Patterns of *crtRB1*-*3’TE*-*1* allele in the CML171 population. (TIFF 419 kb)

Supplementary Fig. 4 Detection of the favorable alleles *crtRB1*-*5’TE*-*2* and *crtRB1*-*3’TE*-*1* in the BC_2_F_1_ generation. (A) Patterns of *crtRB1*-*5’TE*-*2* allele in the CML161 population, (B) Patterns of *crtRB1*-*3’TE*-*1* allele in the CML161 population, (C) Patterns of *crtRB1*-*5’TE*-*2* allele in the CML171 population, (D) Patterns of *crtRB1*-*3’TE*-*1* allele in the CML171 population. (TIFF 392 kb)

Supplementary Fig. 5 Detection of the favorable alleles *crtRB1*-*5’TE*-*2* and *crtRB1*-*3’TE*-*1* in the BC_2_F_2_ generation. (A) Patterns of *crtRB1*-*5’TE*-*2* allele in the CML161 population, (B) Patterns of *crtRB1*-*3’TE*-*1* allele in the CML161 population, (C) Patterns of *crtRB1*-*5’TE*-*2* allele in the CML171 population, (D) Patterns of *crtRB1*-*3’TE*-*1* allele in the CML171 population. (TIFF 284 kb)
