# Supplementary figures and images for: Introgression of the crtRB1 gene into quality protein maize inbred lines using molecular markers
Source: Mol Breed. 2015 Jul 16;35(8):154. doi: 10.1007/s11032-015-0349-7 (PMC4503869; doi:10.1007/s11032-015-0349-7)

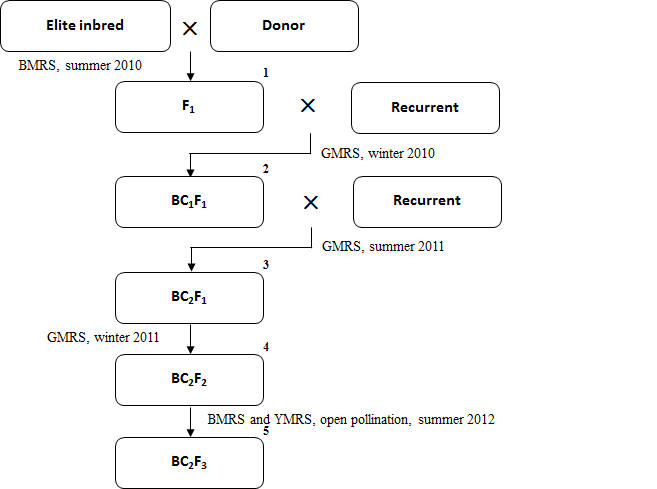

Supplement: Supplementary file 1 — General scheme for the development of backcross and selfed progenies. The temperate maize inbred line Hp321-1 with favorable alleles crtRB1-5’TE-2 and crtRB1-3’TE-1 for high ProVA concentrations was used as male and donor parent. The tropical QPM maize inbred lines CML161 and CML171 were used as female and recurrent parents. The numbers 1, 2, 3, 4, and 5 refer two populations developed for CML161 and CML171, respectively. (TIFF 121 kb) [file 11032_2015_349_MOESM1_ESM.tif]

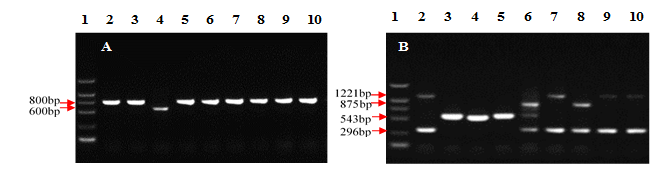

Supplement: Supplementary file 2 — Electrophoresis pattern of functional markers between parents for target alleles. (A) Patterns of crtRB1-5’TE-2 allele; (B) Patterns of crtRB1-3’TE-1 allele; 1, DNA marker; 2, Hp321-272; 3, Hp321-3; 4, Hp321-1; 5, Sc55; 6, A619; 7, By804; 8, CML161; 9, CML171; 10, P138. Lanes 2–7 are maize inbred lines with higher ProVA concentration and favorable LcyE and crtRB1 allelic combination. Lanes 8–10 are maize inbred lines with lower ProVA concentration and unfavorable LcyE and crtRB1 allelic combination. (TIFF 195 kb) [file 11032_2015_349_MOESM2_ESM.tif]

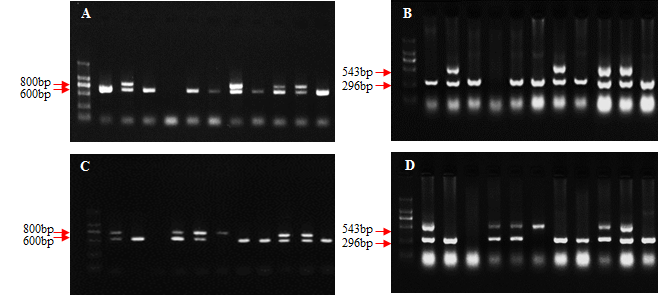

Supplement: Supplementary file 3 — Detection of the favorable alleles crtRB1-5’TE-2 and crtRB1-3’TE-1 in BC1F1 generation. (A) Patterns of crtRB1-5’TE-2 allele in the CML161 population, (B) Patterns of crtRB1-3’TE-1 allele in the CML161 population, (C) Patterns of crtRB1-5’TE-2 allele in the CML171 population, (D) Patterns of crtRB1-3’TE-1 allele in the CML171 population. (TIFF 419 kb) [file 11032_2015_349_MOESM3_ESM.tif]

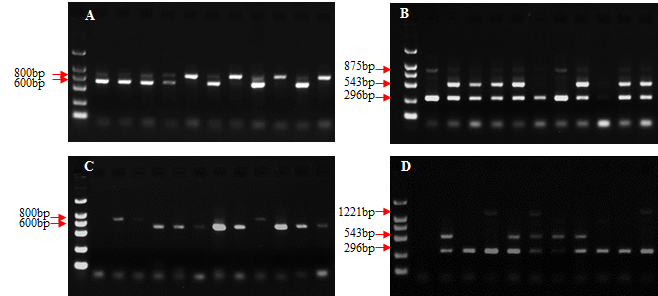

Supplement: Supplementary file 4 — Detection of the favorable alleles crtRB1-5’TE-2 and crtRB1-3’TE-1 in the BC2F1 generation. (A) Patterns of crtRB1-5’TE-2 allele in the CML161 population, (B) Patterns of crtRB1-3’TE-1 allele in the CML161 population, (C) Patterns of crtRB1-5’TE-2 allele in the CML171 population, (D) Patterns of crtRB1-3’TE-1 allele in the CML171 population. (TIFF 392 kb) [file 11032_2015_349_MOESM4_ESM.tif]

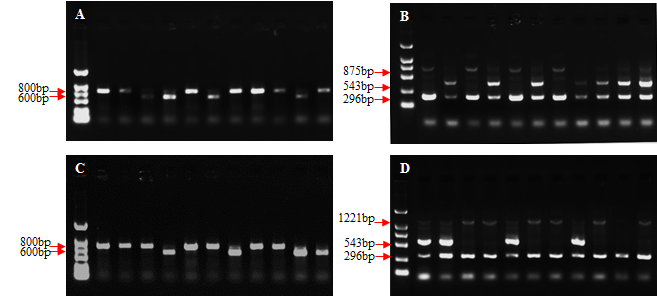

Supplement: Supplementary file 5 — Detection of the favorable alleles crtRB1-5’TE-2 and crtRB1-3’TE-1 in the BC2F2 generation. (A) Patterns of crtRB1-5’TE-2 allele in the CML161 population, (B) Patterns of crtRB1-3’TE-1 allele in the CML161 population, (C) Patterns of crtRB1-5’TE-2 allele in the CML171 population, (D) Patterns of crtRB1-3’TE-1 allele in the CML171 population. (TIFF 284 kb) [file 11032_2015_349_MOESM5_ESM.tif]
